# Validation of segmented and real-time EPI phase contrast flow quantification against segmented gradient echo sequences

**DOI:** 10.1186/1532-429X-17-S1-O66

**Published:** 2015-02-03

**Authors:** Gergely V Szantho, Stephen Lyen, Chris B Lawton, Chiara Bucciarelli-Ducci, Nathan E Manghat, Peter J Weale, Ning Jin, Mark Hamilton

**Affiliations:** 1 Bristol Heart Institute, Bristol, UK; 2 Siemens Healthcare, Camberley, UK

## Background

MR based Flow Quantification is an established method for measuring cardio-pulmonary haemodynamics, but is challenging to perform in situations where it may be advantageous to asses real-world physiological behavior, for example in exercise. The availability of a new real time flow quantification sequence presents the opportunity to measure velocity and flow without these challenges. This study is a preliminary validation of the method as a precursor to use of the sequence in subjects undergoing assessment of induced haemodynamic change by exercise and preload alteration.

## Methods

An Echo Planar flow quantification sequence (Work In Progress package #720 Siemens Healthcare, Erlangen,Germany) was used and validated against commercial flow quantification sequences on a 1.5T MR system (Magnetom Avanto). The sequence was used in two protocols, a segmented version and a real time varient and compared to a standard product segmented protocol.

We measured ascending aortic flow with segmented EPI and RT EPI sequences in 12 and 9 healthy volunteers, respectively, and compared findings to standard flow images. The different sequences were used immediately after one another in each volunteer, during free breathing at rest.

Segmented EPI parameters: TR 17.6ms, TE 5.7ms, Voxel size 1.7x2x10mm, Flip angle 30°, Segments 4.

RT EPI parameters: TR 61ms, TE 5.7ms, Voxel size 2.5x3.2x10mm, Flip angle 30°, Phases 163 (=9.943s to include a full breathing cycle).

Standard flow parameters: TR 29.9ms, TE 2.18ms, Voxel size 1.3x1.3x5mm, Flip angle 30°, Segments 3.

Images were analyzed with commercial flow analysis software (Argus, Siemens Healthcare). RT EPI results were expressed as the mean of the beat-to-beat analysis of the actual acquisition.

We used students' paired t-test, standard deviation and Pearson's correlation coefficient for data presentation.

## Results

All acquired images were evaluable. We found that stroke volume and peak flow rate measured with both segmented and RT EPI were not significantly different from standard flow measurements, showing good correlation with standard stroke volume and peak flow rate.

Segmented EPI versus standard stroke volume: 103.74 +-22.02ml vs 105.2 +-23.96ml, r=0.971, p<0.0001.

RT EPI vs standard stroke volume: 97.74 +-21.48ml vs 105.2 +-23.96ml, r=0.909, p<0.0005.

Segmented EPI vs standard peak flow rate: 499.08 +-91.66ml/s vs 486.19 +-93.11ml/s, r=0.935, p<0.0001

RT EPI vs standard peak flow rate: 482.16 +-85.75ml/s vs 486.19 +-93.11ml/s, r=0.81, p<0.005.

## Conclusions

Real-time and segmented EPI sequences provide reliable aortic stroke volume and peak flow rate at rest. The quick acquisition time and - in the case of the RT EPI sequence - the independency from gating make them a potential tool for detecting physiological changes during pre-load alteration and exercise.

## Funding

The WIP sequences were provided by Siemens Healthcare to our Institution for research purposes. This contract had no financial implications, and none of the parties involved (including Siemens, our Institution and the researchers) received any financial support or reward.

The time of the researchers and the scanner was funded by our Institution and the University.

There is nothing else to disclose.

